# Human Placental Endothelial Cell and Trophoblast Heterogeneity and Differentiation Revealed by Single-Cell RNA Sequencing

**DOI:** 10.3390/cells12010087

**Published:** 2022-12-25

**Authors:** Han Li, Hao Peng, Wei Hong, Yingying Wei, Haojun Tian, Xiaojie Huang, Linyan Jia, Jing Zheng, Tao Duan, Qizhi He, Kai Wang

**Affiliations:** 1 Clinical and Translational Research Center, Shanghai Key Laboratory of Maternal Fetal Medicine, Shanghai Institute of Maternal-Fetal Medicine and Gynecologic Oncology, Shanghai First Maternity and Infant Hospital, School of Medicine, Tongji University, Shanghai 201204, China; 2 Department of Obstetrics and Gynecology, University of Wisconsin-Madison, Madison, WI 53715, USA; 3 Department of Obstetrics, Shanghai First Maternity and Infant Hospital, School of Medicine, Tongji University, Shanghai 201204, China; 4 Department of Pathology, Shanghai First Maternity and Infant Hospital, School of Medicine, Tongji University, Shanghai 201204, China

**Keywords:** human placenta, scRNA-seq, trophoblast, endothelial cell, pregnancy

## Abstract

Background: The placenta is an important organ for fetal and maternal health during pregnancy and impacts offspring health late in life. Defects in placental vasculature and trophoblast have been identified in several pregnancy complications. Thus, the detailed molecular profile and heterogeneity of endothelial cells and trophoblasts in placentas will aid us in better understanding placental behaviors and improving pregnancy outcomes. Methods: Single-cell RNA sequencing (scRNA-seq) was performed to profile the transcriptomics of human placental villous tissues from eleven patients with normal pregnancies in the first and second trimesters (6–16 weeks of gestation). Results: The transcriptomic landscape of 52,179 single cells was obtained, and the cells were classified as trophoblasts, fibroblasts, endothelial cells, erythroid cells, Hofbauer cells, and macrophages. Our analysis further revealed the three subtypes of placental endothelial cells, with distinct metabolic signatures and transcription factor regulatory networks. We also determined the transcriptomic features of the trophoblast subpopulations and characterized two distinct populations of progenitor cells in cytotrophoblasts, which were capable of differentiating to extravillous trophoblasts and syncytiotrophoblasts, respectively. Conclusions: Our study provided a high-resolution molecular profile of the human placenta between 6 and 16 weeks of gestation. Our data revealed the placental cell complexity and demonstrated the transcriptional networks and signaling involved in placental endothelial and trophoblast differentiation during early pregnancy, which will be a resource for future studies of the human placental development.

## 1. Background

The normal growth and development of the placenta are crucial to fetal and maternal health [1]. Functionally, the placenta is involved in nutrients and oxygen transport, protection from mechanical and pathogenic insults, modulation of maternal tolerance, and hormone production [2,3,4]. In fact, placental dysfunction can contribute to severe gestational complications, such as preeclampsia, intrauterine growth restriction, and placenta accretion [5,6,7]. Perez-Garcia, V et al. [8] found that 68% of embryonic lethal mouse knockout lines that are lethal at or after mid-gestation exhibited placental dysmorphologies, highlighting the importance of placental defects in contributing to abnormal embryonic development.

The functional unit of a human placenta is the chorionic villus, which comprises a mesenchymal core and two layers of epithelial trophoblast that cover the surface of the villous tree. The multinucleated syncytiotrophoblasts (SCTs) serve as the outer layer, and the villous cytotrophoblasts (VCTs) form the inner layer. The VCTs also develop to produce a multilayered cellular shell and columns of extravillous trophoblasts (EVTs) that make contact with the decidua [1,9]. Due to the presence of EVT plugs in the uterine spiral arteries, intervillous space O_2_ is as low as 2% before 8-10 weeks of gestation [10,11]. The dissolution of the EVT plugs at the end of the first trimester allows maternal blood delivery into the placenta and the oxygen levels sharply increase 3-fold to approximately 8% [10,11]. To ensure optimal placental function and embryonic development, placental blood vessels must be established and maintained [12]. Defects in placental vasculature are a common placental pathology that have been identified in several pregnancy complications [13,14,15].

The study of human placental development is challenging due to ethical considerations, but the emergence of new technologies has greatly improved our understanding in recent years. The human trophoblast stem cells (TSC) have been established from human blastocysts [16], from the first trimester human placenta in a two-dimensional model [16] and a three-dimensional organoid model [17,18], and by reprogramming naïve pluripotent stem cells [19,20,21]. Additionally, single-cell sequencing, spatial transcriptomics, and mass spectrometry can enhance our understanding of the genetic and epigenetic regulation of placental development [22]. Recent studies have used single-cell RNA sequencing (scRNA-seq) to examine the maternal-fetal interface in the first trimester of pregnancy [23,24], as well as the cellular heterogeneity of placentas from healthy and complicated pregnancies including pre-eclampsia [25,26], preterm [27], and gestational diabetes mellitus [28] during the second and third trimesters. Two studies have characterized trophoblast cell subtypes in villous chorion [29] and smooch chorion [30]. These studies created a map of the human placental transcriptome under both normal and pathological conditions. However, little effort has been made to identify and analyze the molecular profile and heterogeneity of human placental endothelial cells (ECs), possibly due to the limited number of samples or a difference in analytic objectives from the studies mentioned above. Thus, more details remain to be refined in the study of the placenta.

Here, we used scRNA-seq to profile the transcriptome heterogeneity of endothelial cell and trophoblast cell subpopulations throughout the first and second trimesters (6-16 gestational weeks) of the normal human placenta. Our data reveals the transcriptional networks and signaling involved in placental vascularization, as well as complements the transcriptional signatures of trophoblast subtypes responsible for EVT or SCT differentiation. We also found that 8-10 weeks of gestation is a dividing line for placental developmental status. Our research is the first study to describe placental vascular endothelial cell heterogeneity using scRNA-seq. Additionally, the gestational weeks are mostly focused on three stages in the published studies on the human placenta (6-10 weeks, 24 weeks, and 32-40 weeks). We first provided placental scRNA-seq data from the end of the first trimester to the middle of the second trimester (10-16 gestational weeks), when important physiological events, such as elevated partial pressure of oxygen occurred. These findings could serve as useful references for studies of placental development.

## 2. Materials and Methods

### 2.1. Human Placenta Sample Collection

Eleven healthy placental samples were collected from elective terminations between 6 and 16 weeks of gestation at Shanghai First Maternity and Infant Hospital. Gestational age was determined by fetal ultrasound and last menstrual period. We chose 6-16 weeks of gestation as the collection time range because fetal heartbeat can be seen by ultrasound from approximately the 6th week of gestation, and abortions after the 16th week of gestation are carried out with intra-amniotic injections of ethacridine lactate in clinical practice, which could induce different degrees of bleeding, degeneration, and necrosis of the placental tissue, potentially affecting the sequencing results.

### 2.2. Single Cell Dissociation

Placental chorionic villi were identified microscopically. Placental villi from the first-trimester placenta were separated from the chorionic membrane, villi from the second-trimester placenta were separated at the base of villi near the chorionic plate, and then the decidua and chorionic membrane were removed from the villous tissue. The remaining freshly floating and anchoring chorionic villi were washed with cold phosphate-buffered saline (PBS) 3–5 times and then cut into approximately 2–4 mm pieces. Tissues were mechanically and enzymatically dissociated using a human tissue dissociation kit (Miltenyi Biotec, Bergisch Gladbach, Germany, #130-095-929) on a gentleMACS™ Dissociator (Miltenyi Biotec). After dissociation, the homogenate was filtered using a 70-μm cell strainer (Miltenyi Biotec, #130-098-462) and a 40-μm cell strainer (Corning, Coring, NY, USA, #352340). To remove red blood cells, Red Blood Cell Lysis Solution (Miltenyi Biotec, #130-094-183) was used. Subsequently, the cells were washed once and resuspended in 100 μL of Ca^2+^ and Mg^2+^ free PBS containing 0.04% bovine serum albumin (BSA). Cell concentration and cell viability were determined by a hemocytometer and trypan blue staining.

### 2.3. scRNA-seq Data Processing, Quality Control, and Analysis

Single-cell transcriptome profiling was carried out using the Chromium Single Cell instrument (10× Genomics, Pleasanton, CA, USA). The single-cell capturing, barcoding, and cDNA library construction were performed using the single-cell 3′ mRNA kit (V2; 10× Genomics) according to the manufacturer’s protocol. Then, a library quality test was evaluated on the Agilent Bioanalyzer (Agilent Technologies, Santa Clara, CA, USA). The libraries were sequenced with an Illumina NovaSeq 6000 System (Illumina, San Diego, CA, USA, read length: 150 bp, paired end).

CellRanger (10× Genomics) was used to generate digital expression matrices from the FASTQ files obtained from the Illumina sequencing runs. To assure the quality of the downstream analyses, in addition to the filtering of low-quality barcodes (cells), we applied stringent criteria by removing cells with small library size (<1000 UMI) or low transcriptional complexity (Shannon diversity index < 3, calculated by diversity function of the Vegan package in R) from further analysis. The digital expression matrices for cells that passed the quality control were then inputted into the Seurat package in R (v3.0, R Foundation for Statistical Computing, Vienna, Austria) [31,32] to generate Seurat objects for the comprehensive downstream analyses and visualization. The following Seurat functions were used: NormalizeData and ScaleData were used to calculate comparable expression values. FindVariableFeatures was used to identify the variable genes that contribute to the overall similarity/variability of cellular transcriptomic profiles. Seurat RunPCA was used to perform PCA. RunTSNE and RunUMAP were used to calculate the dimensionality reduction coordinates and visualize the result. FindNeighbors and FindClusters were used to perform unsupervised clustering. VlnPlot was used to make violin plots. The functional enrichment analysis was performed using the enricher function of the clusterProfiler package of R.

### 2.4. Cell Type Identification

To identify the cell types and subtypes that in the samples contain, the supervised learning algorithm, SuperCT was used for the preliminary prediction, this algorithm was described in the original paper [33]. The training dataset for SuperCT used in this paper was based on HCL annotation [34]. We also performed the unsupervised clustering using the default parameters to define the putative cell populations. These putative cell populations were cross compared with the SuperCT prediction, and the cell identities were eventually annotated by the unsupervised cell clusters and their majority predicted types.

### 2.5. Cluster Visualization

The relationships across the annotated cell populations were visualized using the hclust function in R based on a self-defined distance metric. The distance metric is determined as follows. Top 10 principal components (PCs) of each cell were calculated using RunPCA of Seurat package. The mean values of the PCs of each cell population were then calculated. These PCs were inputted to calculate the cosine distance, which is implemented by the coop package in R. The cosine distance matrix across the populations was used to plot the clusters.

### 2.6. Analysis of Sub-Clusters within Endothelial Cells and Trophoblasts

We first performed the unsupervised clustering using the FindClusters function of the Seurat Package on the designated cell lineages. We found endothelial cell clusters were largely divided into three distinct classes. We annotated Endo-2 as endovascular progenitor cells based on canonical marker genes and gene sets documented in the previous literature [35]. Another two sub-clusters were annotated according to their biological processes and the gestational period of their existence.

We annotated trophoblasts based on marker genes of SCT (ERVFRD-1, LGALS16, GDF15), EVT (HLA-G, ASCL2), and VCT (KRT7, PARP1, and no expression of EVT and SCT markers). Then all trophoblasts were intuitively subclustered into more detailed cell populations. We carefully examined the similarity of these sub-clusters, merged several sub-clusters based on the similarity of their gene expression patterns. VCT and EVT sub-clusters were annotated according to their biological processes. For the secondary annotation, we used the syncytin-related gene set documented in the previous literature [36,37,38] and dissimilarity analysis to annotate the SCT progenitor cluster. We annotated the EVT progenitor cluster through identifying a putative signature (e.g., epithelial to mesenchymal transition, etc.) and then determined other genes associated with such a signature were also highest expressed in this phenotype.

### 2.7. Pseudotime Trajectory Analysis

To investigate the temporal relationships among the identified clusters, pseudotime analysis was performed using the Monocle 2.0 R package [39]. Genes that were differentially expressed across PhenoGraph-identified clusters were used as an input for the Monocle analysis. For the heatmap representation of pseudotime genes, a time trace of each gene was taken using the “plot_genes_in_pseudotime” function with time divided into 100 equally sized bins. Time was measured by selecting the longest path through the trajectory plot going from t = 0 to t = max. The pseudo-time values of each cell and the gene expression values for that cell were used to fit a linear model using the R function ‘lm’.

### 2.8. RNA Velocity Analysis

RNA velocity was calculated based on the spliced and unspliced counts as previously reported [40], and velocity fields were projected onto the UMAP space of endothelial cells. We used the velocyto.py (v. 11.2) annotator for each BAM file processed by CellRanger using the default parameters. RNA velocity was estimated using a gene-relative model with k-nearest neighbor cell pooling (kCells = 25). Parameter deltaT was set to 1, and fit.quantile was set to 0.02.

### 2.9. Cell-Cycle Analysis

Cell cycle status was determined by using validated genes previously shown to identify cells in active cell cycling phases [41]. Cells that have high scores in either G2/M or S phase-specific gene signature expression were gated and counted based on single-cell RNA sequencing data. The fraction of cells that were in either G2/M or S phase for each cell type was then plotted in a box plot.

### 2.10. Metabolic Gene Expression Analysis

The pseudobulk expression matrix was generated based on the TPM-like value of each cell subpopulation. TPM = copy number for the target gene in a subpopulation × 1M / total copy number of total genes in the same subpopulation. We used Z-values to rescale the expression values in order to show a better contrast between the samples on the heatmap.’pheatmap’ function was used to generate the heatmap. The glycolysis gene set and lipid metabolism gene set were selected from previous studies [42,43]. Nucleotide metabolism gene set, tricarboxylic acid (TCA) gene set, and oxidative phosphorylation (OXPHOS) gene set were selected from the Molecular Signatures Database (MSigDB version 5.2, Broad Institute, Inc., Massachusetts Institute of Technology, and Regents of the University of Calofornia, Cambridge, MA, USA; downloaded 10 June 2020 from http://bioinf.wehi.edu.au/software/MSigDB/).

### 2.11. Gene Set Variation Analysis

Gene set variation analysis (GSVA) was performed in the GSVA R-package (version 1.26.0) [44]. The metabolic gene sets used for analysis were selected from the Molecular Signatures Database (MSigDB version 5.2, Broad Institute, Inc., Massachusetts Institute of Technology, and Regents of the University of Calofornia, Cambridge, MA, USA; downloaded 10 June 2020 from http://bioinf.wehi.edu.au/software/MSigDB/). Pathways with an adj. *p*-value > 0.05 were not considered for further analysis. The upregulated and downregulated pathways were visualized by a heatmap.

### 2.12. Identification of TFs Using SCENIC

After arranging the input from the gene expression matrix, we predicted transcription factor regulons using SCENIC [45] as described above, the RcisTarget package was used to analyze the transcription factor potential binding motifs, and the AUCell package was used to calculate the regulon activity scores of each cell. The heatmap and ggplot2 packages in R were adopted to visualize the expression profile of transcription factors.

### 2.13. Cell-Cell Communication Analysis

CellPhoneDB (version 1.1.0, https://github.com/Teichlab/cellphonedb) was used to predict cell-cell interactions between different cell types [46].

### 2.14. Differentially Expressed Genes between Development Stages

In order to better distinguish the differences between the early and intermediate stages, we performed pseudobulk differential expression profiles between 6 and 7 weeks of gestation (samples D1, D2, and D3) and 14 and 16 weeks of gestation (samples D9, D10, and D11) for endothelial cells, VCTs, EVTs, macrophages, and Hobauer cells. EdgeR was used to perform differential expression analysis [47]. Clusters having fewer than 200 cells from each stage were excluded from this analysis. Metascape [48] was used for functional enrichment analysis.

### 2.15. Immunofluorescence

For immunohistochemical staining, the 6 weeks, 8 weeks, 14 weeks, and 16 weeks of gestation placental samples were fixed in 4% paraformaldehyde, embedded in paraffin, and then sectioned. Multiplex staining and multispectral imaging were used to identify the cell subclusters in the placenta. Multiplex immunofluorescence staining was obtained using the PANO 7-plex IHC kit, cat 0004100100 (Panovue, Beijing, China). Antibodies used for immunohistochemical staining: anti-CD31 (Proteintech, Rosemont, IL, USA, 66065-2-Ig, 1:200), anti-VEGFC (Proteintech, 22601-1-AP, 1:200), anti-CHCHD10 (Proteintech, 25671-1-AP, 1:100), anti-CTHRC1 (Proteintech, 16534-1-AP, 1:200), anti-Ki67 (Abcam, Cambridge, MA, USA, ab16667, 1:200), anti-HLA-G (Cell Signaling Technology, Danvers, MA, USA, #79769, 1:200), anti-EGFR (Abcam, ab32077, 1:200), anti-CLIP1 (Abcam, ab61830, 1:100), anti-TAGLN (Proteintech, 10493-1-AP, 1:200), anti-C1QA (Proteintech, 67063-1-AP, 1:200). CD31 was used as endothelial cell marker, EGFR was used as a trophoblast marker [49,50], HLA-G was used as an EVT marker, and Ki67 was used for mitotic cells. Different primary antibodies were sequentially applied, followed by horseradish peroxidase-conjugated secondary antibody incubation and tyramide signal amplification. Nuclei were stained with 4′-6′-diamidino-2-phenylindole (DAPI, Sigma-Aldrich, St. Louis, MO, USA, D9542) after all the human antigens had been labeled. The stained slides were scanned using the Mantra System (PerkinElmer, Waltham, MA, USA).

### 2.16. Cell Culture

The BeWo cell line was donated by Dr Yuanhui Jia of the Shanghai Key Laboratory of Maternal Fetal Medicine, Shanghai First Maternity and Infant Hospital of Tongji University. BeWo cells were cultured in F-12K medium (Sigma-Aldrich) supplemented with 10% fetal bovine serum (Gibco, Grand Island, NY, USA), 1% penicillin/streptomycin (Gibco, Grand Island, NY, USA) at 37°C, 5% CO_2_. Cells were treated with 50 μM forskolin (Beyotime, Shanghai, China) for 48 h, which is commonly used to stimulate syncytialization.

### 2.17. Western Blot Analysis

The protein concentration was quantified using the Pierce BCA Protein Assay Kit (Thermo Fisher Scientific, Waltham, MA, USA) following the manufacturer’s instructions. Proteins were separated by 10% SDS-PAGE gels and transferred to PVDF membranes by gel electrophoresis and electroblotting, respectively. After blocking with 5% BSA, blots were probed with primary antibodies at 4°C overnight. Then, membranes were washed and incubated with secondary antibodies. Ultimately, proteins were visualized using the enhanced chemiluminescence reagents (Thermo Fisher Scientific). The antibodies we used were: anti-CLIP1 (Abcam, ab61830, 1:1000), anti-TBX3 (Abcam, ab99302, 1:1000), anti-ERVFRD-1 (Abcam, ab230235, 1:1000), anti-GAPDH (Cell Signaling Technology, #5174, 1:2000), anti-rabbit-IgG (Cell Signaling Technology, #7074, 1:5000), and anti-mouse-IgG (Cell Signaling Technology, #7076, 1:5000).

### 2.18. RNA Isolation and Quantitative RT-PCR

Total RNA was extracted using TRIzol (Invitrogen, Carlsbad, CA, USA); RNA was then reverse transcribed using the PrimeScript RT reagent kit (Takara, Japan). Quantitative RT-PCR (qRT-PCR) was performed using the SYBR Green PCR master mix (Takara, Japan) and the StepOnePlus PCR system (Thermo Fisher Scientific) according to the manufacturer’s instructions. Relative mRNA levels were calculated using the 2-ΔΔCT method and presented as the fold change relative to the control. The primers are shown in Table S1.

### 2.19. Statistical Analysis

Data were expressed as mean with SD of at least three independent times, and the unpaired Student’s *t* tests were used to determine statistical significance. A *p*-value < 0.05 was considered statistically significant.

## 3. Results

### 3.1. Single-Cell Transcriptome Profiling of Normal Human Placentas in the First and Second Trimesters

**Figure 1:**
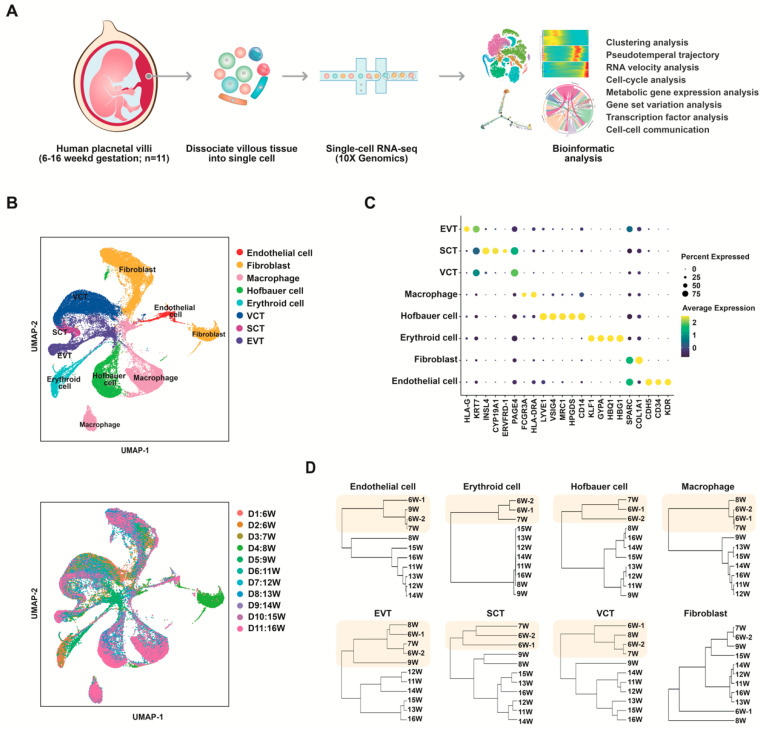
Single-cell RNA-seq of human placenta samples at 6–16 weeks of gestational age. (**A**) Overview of the experimental workflow. (**B**) UMAP plots of cells from human placenta samples, colored by cell type (top) and tissue origin (bottom). (**C**) Dot plot showing the selected marker genes in all cell types. (**D**) Dendrogram visualization of unsupervised hierarchical clustering analysis of expression phenotypes showing the relationships of cells sampled at different gestational ages.

We collected 11 fresh and healthy placental villi samples from elective terminations between 6 and 16 weeks of gestational age (6–16 W), when the majority of dynamic placental development has not yet been completed. The placental villi samples we used for sequencing included cells isolated from floating and anchoring villi and areas surrounding the cell column, while most of the decidua were removed. The tissues were processed and dissociated into single-cell suspensions and sequenced using the 10× Genomics scRNA-seq platform (Figure 1A). We obtained high-quality transcriptomic profile data from a total of 52,179 cells that passed stringent quality control (total UMI count > 1000; transcriptional complexity > 3). The clinical information and quality control data for all samples are provided in Table S2 and Figure S1A.

We defined eight major cell types: trophoblasts (EVTs, SCTs, and VCTs), macrophages, Hofbauer cells, erythroid cells, fibroblasts, and endothelial cells (Figure 1B,C). Cell type-specific signature genes (Table S3, Figure S1B) and the number of cells analyzed from each sample per cluster (Table S4, Figure S1C) are listed. As trophoblasts are present only in the placenta and not in maternal tissues, the sex of each sample was inferred from the expression of *RPS4Y1* in trophoblast cells [51] (Figure S1D).

By using hierarchical clustering analysis of transcriptome patterns of each cell type from each developmental stage (Figure 1D), we found similar transcriptome phenotypes between 6 and 7 gestational weeks and between samples after 8–9 gestational weeks in most of the cell types. This finding suggested that changes in cellular gene profiles during placental development are related to developmental time, the period of 8–9 gestational weeks might be a critical time point for altering gene expression profiles in placental cells during early pregnancy.

### 3.2. Identification and Characterization of Distinct Endothelial Clusters in Human Placenta

**Figure 2:**
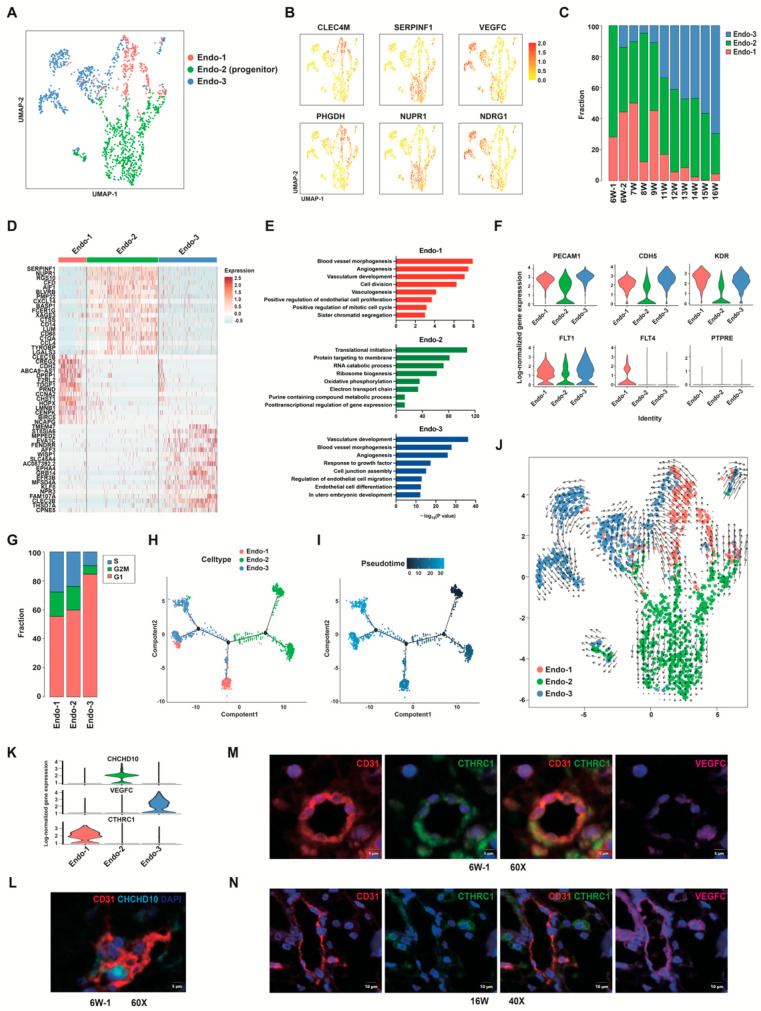
Single-cell RNA-seq analysis of placental endothelial cells from the first and second trimesters. (**A**) UMAP plot showing three different subtypes of human placental endothelial cells. (**B**) UMAP plots of the expression distribution for selected cluster-specific genes. (**C**) The proportions of three EC clusters at each developmental timepoint. (**D**) Heatmap of top 20 DEGs in three EC clusters. (**E**) Representative GO analysis terms of DEGs in Endo-1 (red), Endo-2 (green) and Endo-3 (blue) clusters. (**F**) Violin plots showing the expression levels of endothelial markers *PECAM1* and *CDH5*, VEGFR coding genes *FLT1*, *FLT4*, and *KDR*, hematopoietic marker *PTPRE* in each EC clusters. (**G**) Cell cycle analysis of three EC clusters. (**H**,**I**) Pseudotime analysis of placental endothelial cells, cells on the tree are colored by cluster (**H**) or pseudotime (**I**). (**J**) Projection of non-linear RNA velocity fields onto the UMAP space in (**A**). (**K**) Violin plot displaying selected cluster-specific genes. (**L**) Immunofluorescence staining of human first-trimester placental villi using the CD31 and CHCHD10 antibodies. DAPI staining shows the nuclei. Scale bar, 5 μm. (**M**) Immunofluorescence staining of human first trimester placental villi using CD31, VEGFC, CTHRC1 antibodies. Scale bar, 5 μm. (**N**) Immunofluorescence staining of human second trimester placental villi using CD31, VEGFC, CTHRC1 antibodies. Scale bar, 10 μm. DEGs, differentially expressed genes.

To identify subtypes of placental endothelial cells, second-level clustering was performed. We identified three major EC clusters (Endo-1, -2, and -3) by unsupervised hierarchical clustering analysis (Figure 2A). Representative markers for each cluster are shown in UMAP plots (Figure 2B, Figure S2A). The proportion of the three cell clusters varied throughout the study period (Figure 2C).

Cells in Endo-2 highly expressed a number of genes that were specifically expressed in aortic endovascular progenitor cells [35], including *SERPINF1, NUPR1, PMP22, CXCL14*, *FCER1G*, *CTSS*, *LUM*, *CCL4*, *TYROBP*, *CHCHD10*, *FBLN1*, *VCAN*, *DCN, COL1A1*, *CD74*, *CD36,* and *TIMP2* (Table S5). Differentially expressed genes (DEGs) in Endo-2 were typically involved in translation, peptide metabolic process, and ATP metabolic process (Figure 2D,E). The expression levels of the endothelial markers *PECAM1* and *CDH5*, and VEGFR coding genes *KDR* and *FLT1* in Endo-2 were lower than in Endo-1 and Endo-3 (Figure 2F). All three clusters did not express the hematopoietic marker *PTPRC*, indicating that there was no hematopoietic contamination (Figure 2F). Immature ECs in the aorta were also characterized by the lack of specific marker genes (e.g., *PECAM1* and *CDH5*), but upregulated ribosomal gene expression, consistent with an activated intermediate phenotype [35]. Our results suggested that Endo-2 cells were local vascular progenitor cells or immature ECs in placentas.

Endo-1 cells were present primarily in first trimester placentas and decreased in abundance after 11 weeks of gestation (Figure 2C). The DEGs in Endo-1 were mainly related to blood vessel morphogenesis, vasculogenesis, angiogenesis, and cell division (Figure 2E). Endo-1 cells might be a group of cells undergoing endothelial proliferation and blood vessel formation in the early stages of pregnancy. Interestingly, we found that VEGFR-3 coding gene *FLT4* was mainly expressed in Endo-1 (Figure 2F). VEGFR-3 is involved in early vasculature development in tumors [52] and coronary thrombi [53]. Consistent with previous studies, our results suggest a role for VEGFR-3 in early vascular development in the placenta during the first trimester of pregnancy.

Cells in Endo-3 expressed higher levels of *VEGFC*, *NDRG1*, *FOXP1*, *MEF2A*, and *NOTCH4* (Figure 2B, Figure S2A). The proportion of Endo-3 in endothelial cells increased gradually with the development of gestational age (Figure 2C). In Endo-3, upregulated genes were related to tube morphogenesis, angiogenesis, response to growth factors, regulation of cell migration and cell differentiation (Figure 2E). We found genes related to endothelial cell differentiation (Figure S2B) and migration (Figure S2C) were mainly upregulated in Endo-3. These results suggested the Endo-3 as the main cell type in the second trimester, displayed a more differentiated and mature vascular endothelial signature.

The pseudotime trajectory revealed a continuous of cells with three distinct branch points (Figure 2H). By comparing the cells on the tree colored by cluster (Figure 2H), pseudotime (Figure 2I), and gestation age (Figure S3A), we found the less differentiated side (dark blue in Figure 2I) was mainly composed of Endo-2, while the more differentiated sides (blue in Figure 2I) were occupied by Endo-1 and Endo-3 (Figure 2H). In addition, Endo-1 and Endo-3 were located at discontinuous branches (Figure 2H), the development of these two clusters might not be a simple continuous process. Instead, Endo-1 and Endo-3 may both represent differentiated endothelial cells, and dominate at different gestational ages. Then we performed RNA velocity analysis to predict the relationships between cells based on the proportion of exonic and intronic reads [40]. RNA velocity analysis identified Endo-2 as the root for three differentiation trajectories: self-renewal, differentiation to Endo-1, and differentiation to Endo-3 (Figure 2J). Endo-1 cells also showed directionality and magnitude toward Endo-3. These data further supported the classification of Endo-2 as placental vascular progenitor cells.

Immunofluorescence staining of human placental villi using the endothelial cell marker CD31, and CHCHD10, a molecule that is highly expressed in Endo-1, is shown in Figure 2L. We also selected CTHRC1 for Endo-1 and VEGFC for Endo-3 as markers to distinguish these two subtypes on human first and second trimester placental villi (Figure 2M,N). CTHRC1 was co-expressed with CD31 in the placental vasculature in early pregnancy and was not significantly expressed at 16 weeks of gestation. In contrast, VEGFC was significantly expressed in the placental vasculature at mid-pregnancy (Figure 2N).

### 3.3. Metabolic Transcriptome Signatures in Different Placental Endothelial Clusters

**Figure 3:**
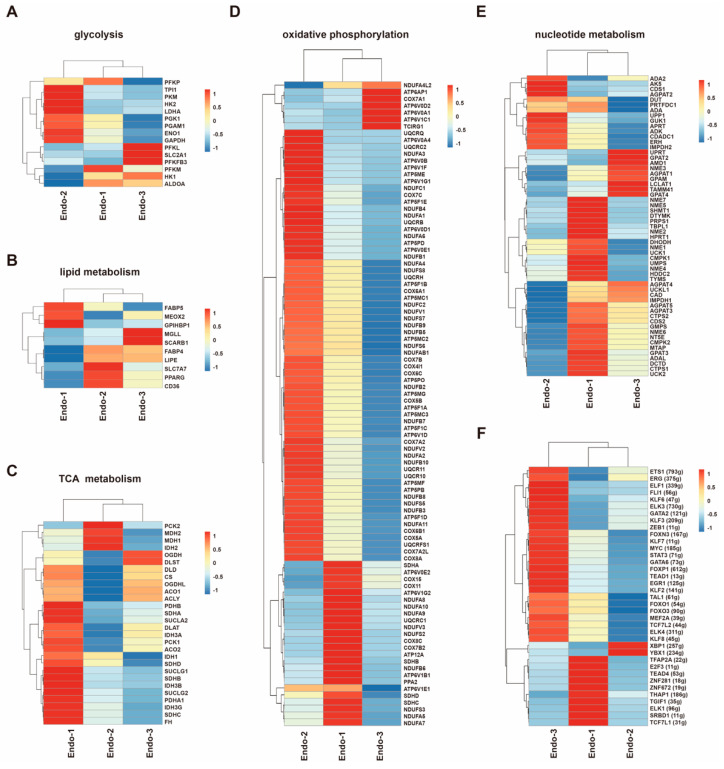
Metabolic heterogeneity and transcriptional regulatory heterogeneity in placental endothelial cells. (**A**–**E**) Heatmaps of representative metabolic genes involved in glycolysis (**A**), lipid metabolism (**B**), TCA metabolism (**C**), oxidative phosphorylation (**D**), and nucleotide metabolism (**E**) in three EC clusters. (**F**) Activity heatmap of the inferred transcription-factor gene-regulatory networks (SCENIC). The t values of AUC scores of expression regulation by transcription factors are estimated using SCENIC, per EC cluster. Color scale: red, high expression; blue, low expression.

In the GSVA analysis, we found that oxidative phosphorylation and glycolysis gluconeogenesis were upregulated in Endo-2 (Figure S2G). TCA cycle, purine and pyrimidine nucleoside biosynthetic process were upregulated in Endo-1 (Figure S2G). Heatmap analysis revealed that three EC clusters exhibited distinct metabolic gene signatures (Figure 3A–E).

Genes involved in glycolysis were mainly upregulated in Endo-2 (Figure 3A). DEGs in Endo-2 were involved in nucleotide biosynthetic process and purine nucleotide metabolic process (Table S5), an increase in glycolysis in Endo-2 could provide the energy for biosynthesis and proliferation. We noted that *PFKFB3*, a gene encoding glycolysis rate-limiting enzyme, was increased in Endo-3 (Figure 3A), supporting the importance of *PFKFB3* in robust placental angiogenesis.

The distribution of lipid metabolism genes in the three endothelial cell clusters was not well defined (Figure 3B). Most of the genes involved in TCA metabolism were increased in Endo-1 (Figure 3C). TCA cycle could produce metabolic intermediates for synthesis of amino acids, nucleotides, and lipids [54], which need to be duplicated during endothelial cell division and necessary for cell growth. Besides TCA cycle, we also found that gene encoding phosphoglycerate dehydrogenase (PHGDH), a key enzyme of the serine synthesis pathway [55], was also upregulated in Endo-1 (Figure 2B). Nucleotide metabolism related genes were also mainly upregulated in Endo-1 (Figure 3E). Thus, Endo-1 cells, which undergo through endothelial differentiation and proliferation in the early stages of pregnancy, may rely on the TCA cycle and anabolic side-pathways, such as serine synthesis pathway, for nucleotide synthesis and amino acid synthesis.

Oxidative phosphorylation gene signatures were mainly upregulated in Endo-2 and Endo-1 (Figure 3D), in line with reports showing the importance of OXPHOS in the development and proliferation of ECs [56]. Endo-3 as the main EC cluster in second trimester, exposed to elevated oxygen partial pressure (at the end of the first trimester placenta oxygen levels increase to approximately 8% [10,11]) compared with Endo-1, but cells in Endo-3 undergo a primarily glycolytic metabolism rather than oxidative glucose metabolism. In summary, these observations indicated that the metabolic demands of placental vascular endothelial cells are subtype or stage dependent.

### 3.4. Transcription Factor Regulatory Network in Different Placental Endothelial Clusters

We used single-cell regulatory network inference and clustering (SCENIC) [45] to investigate the underlying molecular mechanisms driving the differentiation of ECs. The regulon activity score (RAS), regulon specificity score (RSS) and relative expression levels of each transcription factor (TF) are shown in Tables S7 and S8, respectively. This analysis showed that each of the clusters was characterized by a unique transcription factor network. Representative regulons with biological significance and whose regulon activity and transcription factor themselves both upregulated in one cluster were shown in Figure 3F.

Endo-1 had several upregulated regulons involved in transcriptional regulation (Figure 3F), these regulons included *ZNF672* (19 g), *ZNF281* (18 g), *TGIF1* (35 g), *SRBD1* (11 g), *TFAP2A* (22 g), and *E2F3* (11 g). Other upregulated transcription factors and their networks in Endo-1 may also play important roles in early human placental EC differentiation and vascular maturation. For instance, *THAP1* is a DNA-binding transcription regulator that regulates endothelial cell proliferation and G1/S cell-cycle progression [57]. *ELK1* regulates angiopoietin-1 signaling and the angiogenic response [58]. *TEAD4* is involved in controlling EC divide and vascular growth in mice [59].

Many regulons driven by key endothelial transcription factors were enriched in Endo-3, such as *MEF2A*, *ZEB1*, *STAT3*, *TAL1*, *GATA2*, *GATA6*, Krüppel-like factor family (*KLF2*, *KLF3*, *KLF6*, *KLF7*, *KLF8*), Forkhead family (*FOXP1*, *FOXN3*, *FOXO1*, *FOXO3*), and ETS family (*ELF1*, *ELK3*, *ELK4*, *FLI1*, *ERG*, *ETS1*) (Figure 3F). Krüppel-like factor (KLF) family appears to function in endothelial cells after initial specification and differentiation [60]. Forkhead (FOX) family regulate the correct organization of the vascular system [61]. ETS transcription factor family are central regulators of endothelial gene expression [62,63]. These results proved that cells in Endo-3 exhibit an angiogenic phenotype, with the activation of a series of transcription factors related to vascular function.

### 3.5. Subclusters of Placental Trophoblast Cells

**Figure 4:**
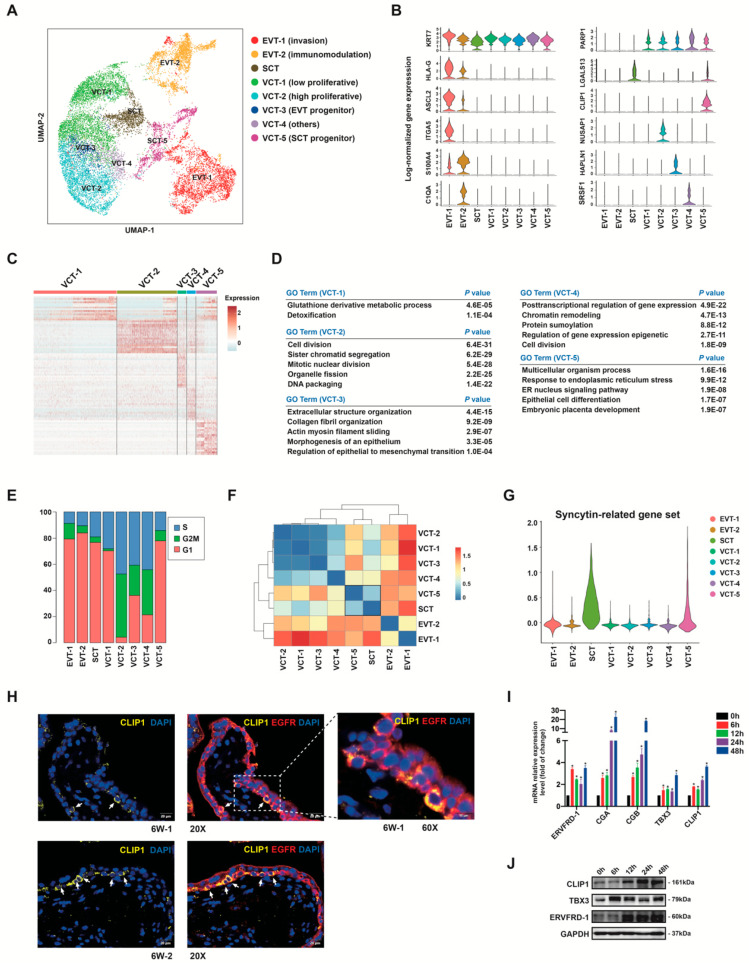
Cell subtype identification of human placental trophoblast cells. (**A**) UMAP plot showing eight subtypes of human placental trophoblast cells. (**B**) Violin plots showing the expression levels of representative marker genes across the eight clusters. *Y*-axis demonstrates log scale normalized read count. (**C**,**D**) Heatmap and GO enriched terms of cell type differentially expressed genes among the five VCT clusters. (**E**) Cell cycle analysis of eight trophoblast clusters. (**F**) Heatmap and the clustering structure show the dissimilarity across the trophoblast subpopulations. Blue color denotes the high similarity and red color denotes the low similarity. (**G**) Violin plots showing the expression levels of a representative syncytin-related gene set (*ERVFRD-1*, *MFSD2A*, *ERVW-1*, *ASCT1*, *ASCT2*) for each trophoblast cluster. (**H**) Immunofluorescence staining for the indicated VCT-5 marker CLIP1 and trophoblast marker EGFR in 6 weeks of gestation placenta. The white arrowheads indicate EGFR^+^CLIP1^+^ cells. Scale bars, 20 μm. (**I**,**J**) In vitro analysis confirmed CLIP1 is induced under fusion differentiation conditions. BeWo trophoblast cells were exposed to differentiating conditions (50 μM forskolin) for 0, 6, 12, 24, or 48 h. qRT-PCR (**I**) and Western blotting (**J**) showed the expression of CLIP1 and SCT marker genes mRNA and protein levels evaluated. * *p* < 0.05, n = 3.

Based on the expression of well-known marker genes, placental trophoblasts can be classified into SCTs, VCTs, and EVTs (Figure S4A). To further reveal heterogeneity among trophoblasts, 16,819 high-quality trophoblasts were re-clustered by unsupervised clustering. After evaluating the similarity of gene expression patterns of the eighteen unsupervised clustering groups, we appropriately merged the subgroups with similar gene expression as appropriate (Figure S4B–D). Eight subgroups were obtained (Figure 4A), including five populations of VCT (VCT-1, -2, -3, -4, -5), two populations of EVT (EVT-1 and -2) and one cluster of SCT. Violin plots revealed that *KRT7* was expressed in all subtypes, *PARP1* as a VCT marker was expressed in all VCT clusters, *HLA-G* and *ASCL2* as EVT markers were expressed in EVT-1 and -2 (Figure 4B), *LGALS13*, *LGALS16* and *ERVFRD-1* were expressed in SCT (Figure 4B, Figure S5A). Beside these common markers, each cluster contained unique marker gene profiles (Figure S4E, Table S9). The proportions of the eight trophoblast subtypes in each sample is shown in Figure S4F.

Heatmap of the top differentially expressed genes (Figure 4C) and GO analysis for each VCT subtype (Figure 4D) were performed. To study the heterogeneity across different clusters, we analyzed the dissimilarity [64] between each cluster (Figure 4F), blue color denotes the high similarity and red color denotes the low similarity. We found high similarity between the gene expression patterns of VCT-5 and SCT (Figure 4F). DEGs in VCT-5 cells were involved in epithelial cell differentiation (Figure 4D). The SCT highly expressed genes *ERVW-1*, *TBX3*, *CYP19A1* were also upregulated in VCT-5 (Figure S5A). As human endogenous retrovirus-encoded envelope protein syncytins are important players in fusion process [65], we explored the expression level of syncytin-related gene set in all trophoblast clusters (Figure 4G). This gene set includes five genes, *ERVW-1* and its receptor gene *ASCT1* and *ASCT2*, *ERVFRD-1* and its receptor gene *MFSD2A* [36,37,38]. We found that syncytin-related genes were selectively expressed in SCT and VCT-5 (Figure 4G), suggesting the fusion-capacity of VCT-5. From these results, we speculated that cells in VCT-5 act as SCT progenitor cells, and that VCT-5 is the cell population that tends to fuse or is early in the process of differentiation towards SCT. *CLIP1* was one of the marker genes that we screened for VCT-5. CLIP1 positive cells were primarily located in the monolayer of villous cytotrophoblasts (Figure 4H, Figure S6C,D). To verify that the expression of CLIP1 changes during the fusion process, we used in vitro trophoblast differentiation system, which modeled VCT to SCT differentiation by treating trophoblast cell line BeWo with forskolin. Expression of fusion related genes ERVFRD-1, CGA, CGB, TBX3 were used as indicators of cell fusion. We found that CLIP1 was induced at both the RNA and protein levels following stimulation of fusion differentiation (Figure 4I,J).

Cells in VCT-5 exhibited the proliferative inactive state (Figure 4E). As we know, when cytotrophoblasts prepare to undergo syncytial fusion, they will leave the cell cycle [65]. Another proliferation inactive cluster VCT-1 (Figure 4E) did not express genes typically associated with differentiation. Thus, VCT-1 might act as the quiescent state population, was not involved in the process of trophoblast proliferation or differentiation.

**Figure 5:**
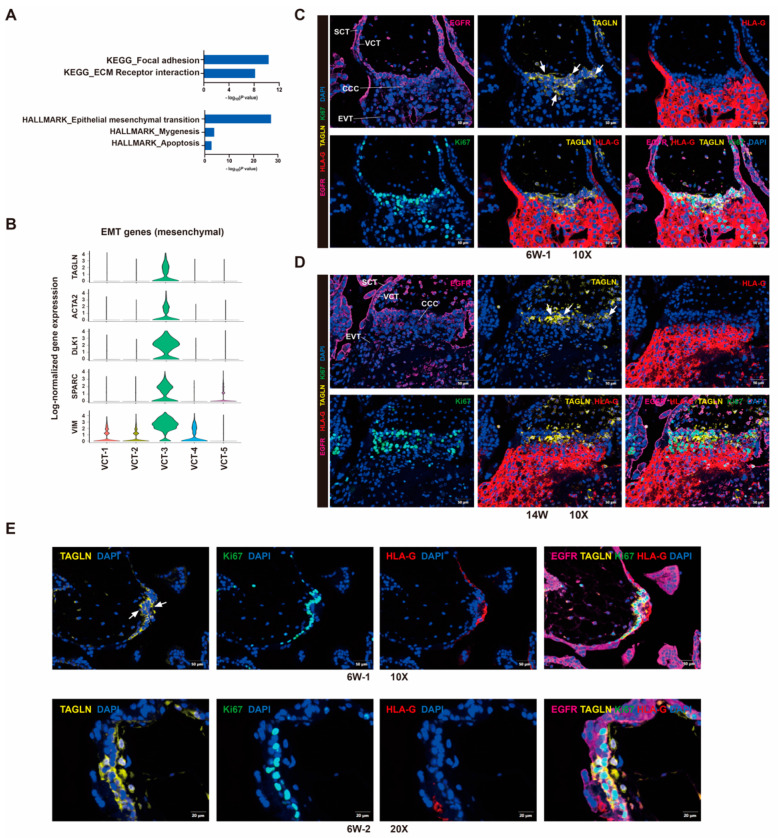
Human trophoblast progenitor cells contribute to EVT lineages. (**A**) Selected top categories from KEGG and Cancer Hallmark analysis of differentially expressed genes in VCT-3. (**B**) Violin plots showing the relative expression of EMT genes in each VCT cluster. (**C**,**D**) Immunofluorescence staining for the indicated VCT-3 marker TAGLN, trophoblast marker EGFR, EVT marker HLA-G, proliferative cell marker Ki67 in cytotrophoblast cell columns structures of 6 weeks of gestation (**C**) and 14 weeks of gestation (**D**) placental tissue. Scale bars, 50 μm (**E**) Immunofluorescence staining for TAGLN in the two-layer cell structure of 6 weeks of gestation placental villi. The white arrowheads indicated VCT-3 cells, which were TAGLN and EGFR double positive, and HLA-G negative. Scale bars, 50 μm for 10× and 20 μm for 20×.

VCT-2 enriched genes were associated with cell division (Figure 4D). Cell cycle analysis indicated that VCT-2 exhibited the highest proliferative activity (Figure 4E). VCT-3 and VCT-4 also expressed the cell cycle-related genes (Figure S5A) and had higher proportion of G2/M stage cells (Figure 5E) than VCT-1 and VCT-5. Ki67 positive cytotrophoblast cells mainly localized in villous cytotrophoblast and in the base of the cytotrophoblast cell columns (CCCs) in early first trimester and second trimester placenta (Figure S6A,B). VCT-3 cells were associated with extracellular structure organization and positive regulation of epithelial to mesenchymal transition (EMT) (Figure 4D), suggesting the early features of the human trophoblast differentiation from VCT to EVT. We hypothesized that VCT-3 cells likely act as trophoblast EVT progenitors contributing to EVT differentiation, which we would discuss in more detail later (Figure 5). Although of interest, the function of VCT-4 remains undetermined, and its relevance to trophoblast biology was unclear.

Taken together, the findings we have discussed so far suggest that in the first and second trimesters, villous cytotrophoblasts are highly heterogeneous. In this regard, we have predicted at least four functionally distinct subtypes of villous cytotrophoblasts: cells that are not in the process of differentiation including inactive proliferating cells (VCT-1) and active proliferating cells (VCT-2) in the villi; cells that enter the process of differentiation including one committed to EVT pathway (VCT-3) and the other committed to syncytialization (VCT-5).

### 3.6. Human Trophoblast Progenitors Contribute to EVT Differentiation

VCT-3, as an active proliferative subpopulation (Figure 4E), had gene expression features of VCT to EVT differentiation, such as cell adhesion, ECM receptor interaction, and epithelial to mesenchymal transition (Figure 4D, Figure 5A). A subset of EMT genes were selectively expressed in VCT-3 among all VCT subtypes (Figure 5B). We selected TAGLN as a marker to distinguish VCT-3 location via immunofluorescence (Figure 5C,D), and used Ki67, EGFR, HLA-G antibodies to stain for mitotic cells, trophoblasts, and EVTs, respectively. TAGLN positive cells were mainly localized at the proximal side of CCCs in placentas at 6 weeks (Figure 5C) and 14 weeks (Figure 5D) of gestation. The majority of TAGLN positive trophoblasts were also positive for Ki67. The distal side of trophoblast cell columns showed extravillous trophoblast phenotype, which were TAGLN negative but HLA-G positive.

We found TAGLN and EGFR double positive areas in several first trimester villi that did not have CCC structures, which were characterized by cell proliferation and stacking, and a slight destruction of the two-layer cell structure of villi (Figure 5E). We hypothesized that these areas were the initial sites of cytotrophoblast cell column establishment and EVT differentiation. In general, we found that cells in VCT-3 acted as mitotically active trophoblast progenitors with the potential to form EVT outgrowth.

### 3.7. VCT to EVT Differentiation Analysis

**Figure 6:**
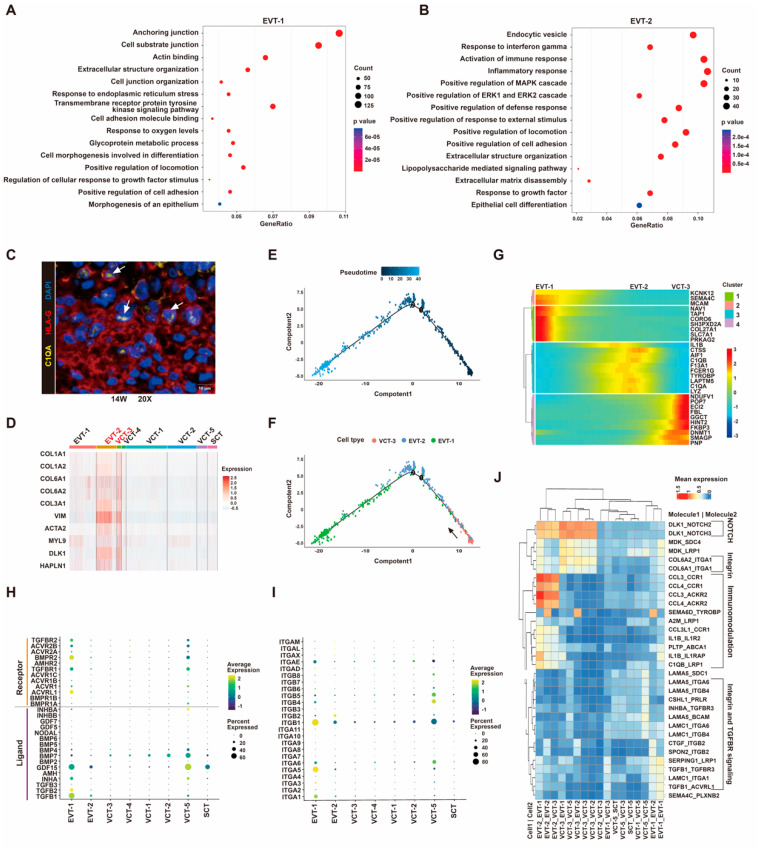
EVT subtypes and VCT to EVT differentiation analysis. (**A**,**B**) GO analysis of differentially expressed genes of EVT1 (**A**) and EVT2 (**B**). The size of the circles represents the number of significantly enriched genes in each item. The color key from blue to red indicates low to high average gene expression. (**C**) Immunofluorescence staining for C1QA and HLA-G in the decidua of 14 gestational weeks human placenta. The white arrowheads indicate C1QA and HLA-G double positive cells. Scale bars, 10 μm. (**D**) Heatmap showing the relative expression of EMT genes in each trophoblast cluster. (**E**,**F**) Pseudotime analysis of VCT-3 and EVTs, cells on the tree are colored by pseudotime (**E**) and cluster (**F**). (**G**) Expression pattern over pseudotime for the top 10 most variable genes were shown on the *y*-axis. Each column represents one cell. Expression ranged from dark blue (lowest) to red (highest). (**H**,**I**) The expression of TGF-β signaling pathway (**H**) and integrins (**I**) in trophoblast clusters. The color key from blue to yellow indicates low to high average gene expression, respectively. The dot size indicates the percentage of cells expressing a certain marker. (**J**) Ligand-receptor interaction analysis within trophoblast clusters. Molecule 1 is expressed by Cell 1 and Molecule 2 is expressed by Cell 2.

In our study, EVTs were mainly isolated from cell columns at the tips of anchoring villi. We clustered EVTs into two populations. DEGs in EVT-1 were related to anchoring junction, extracellular structure organization, regulation of locomotion and adhesion, and integrin cell surface interactions (Figure 6A, Figure S5C). DEGs in EVT-2 were associated with activation of immune response, inflammatory response, metabolism of mRNA and translation (Figure 6B, Figure S5D). We selected C1QA as a marker to distinguish EVT-2 using immunofluorescence staining (Figure 6C, Figure S6E). Similar to VCT-3, EVT-2 also expressed EMT-related genes (Figure 6D). We performed pseudotime analysis to order single cells from VCT-3, EVT-2, and EVT-1, and construct the differentiation trajectory (Figure 6E,F). VCT-3 cells made up the less differentiated origin site, followed by EVT-2 cells, which occupied the right half of the major trajectory, and EVT-1 cells distributed broadly across the pseudotime space (Figure 6E,F). We generated a heatmap to show the gene expression dynamics of the trajectory (Figure 6G). Several common invasion-related genes were differentially expressed in these two EVT clusters (Figure S5B). For instance, *MMP12*, a metalloproteinase implicated in inflammation [66] was expressed in EVT-2 cells. *GCM1*, a transcription factor that stimulates trophoblast migration and invasion [67], was mainly expressed in EVT-1 cells. These results indicated that EVT-2 might represent an early differentiation stage characterized by anti-inflammatory and immunomodulation, low expression of the EVT signature gene and with the presence of the EMT signature. Meanwhile cells in EVT-1 were in a mature stage characterized by the regulation of locomotion, adhesion, and invasion.

We found transforming growth factor beta (TGF-β) receptor signaling pathway and integrin mediated signaling pathway were enriched in EVT-1 and EVT-2 (Table S10). The expression of TGF-β signaling receptors *TGFB1R*, *TGFB2R, BMPR2*, *ACVRL1*, and *ACVR2B* were gradually increased from EVT-2 to EVT-1 (Figure 6H). Several TGF-β signaling ligands such as *TGFB1*, *TGFB2, GDF15*, *INHA* were also upregulated in EVT-1 (Figure 6H). Our finding was consistent with a recent study which reported TGF-β signaling plays a key role in the differentiation program of EVTs [68]. Integrin switching takes place during invasive trophoblast differentiation in vitro [69,70]. In our results, *ITGB1*, *ITGA5*, and *ITGA1* were found to be expressed in EVT-2, and significantly upregulated in EVT-1 (Figure 6I). In addition, *ITGB2* was expressed in EVT-2 (Figure 6I), which is related to inflammatory and immune-related responses [71]. The Notch receptor gene *NOTCH2* and Notch ligands such as *JAG2* were expressed by EVT-1 cells (Figure S5E). We also identified numerous interactions among EVT-1, EVT-2, and VCT-3, including interactions associated with immunomodulation, adhesion, and cell signaling pathways (Notch and TGF beta receptor signaling) (Figure 6J).

### 3.8. Other Cell Types

**Figure 7:**
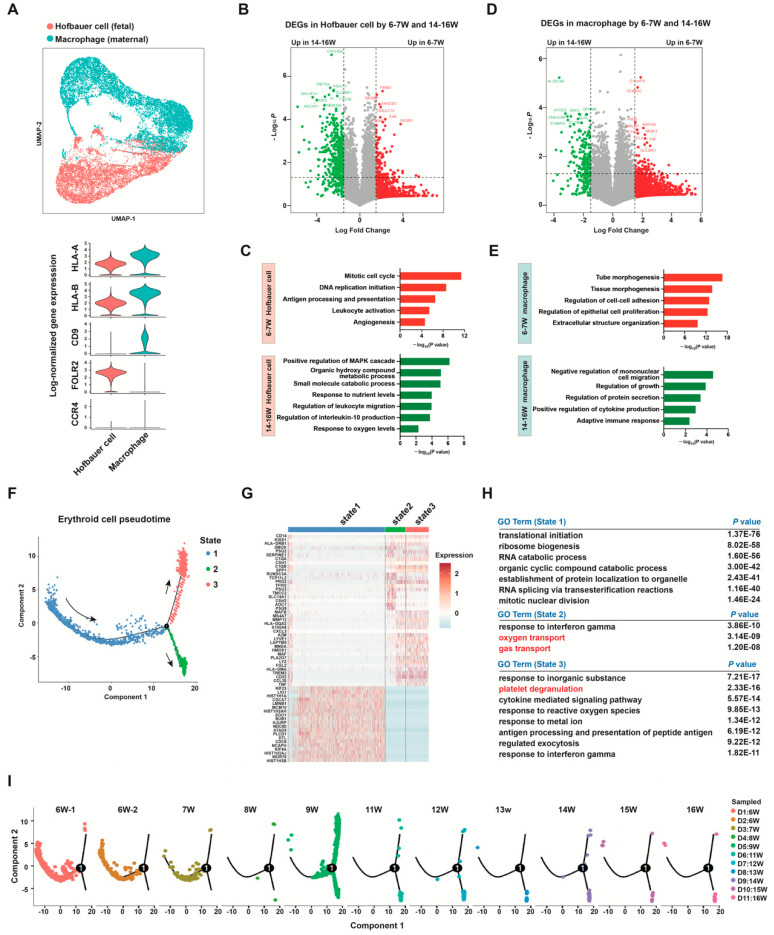
Immune cell and erythroid cell analysis. (**A**) UMAP plot and violin plots of HLA-A, HLA-B, CD9, FOLR2, and CCR4 log-normalized gene expression in Hobauer cells and macrophages. (**B**,**C**) Volcano plot of the DEGs between 6–7 weeks of gestation (6–7 W) and 14–16 weeks of gestation (14–16 W) for Hofbauer cells (**B**) and representative GO analysis terms of these DEGs in Hofbauer cells (**C**). (**D**,**E**) Volcano plot of the DEGs between 6–7 W and 14–16 W for macrophages (**D**) and GO analysis terms of these DEGs in macrophages (**E**). Genes with a log fold change greater than an absolute value of 2 were plotted. Genes with greater expression at 6–7 W are shown in red, and genes with greater expression at 14–16 W are shown in green. (**F**) In a pseudotime analysis of placental erythroid cells, cells on the tree were colored by state. (**G**) Heatmap of the top 20 DEGs of each erythroid state. (**H**) GO terms enriched among the DEGs of the three erythroid states. (**I**) Pseudotime analysis of placental erythroid cells, cells on the tree are colored by sample. DEGs, differentially expressed genes.

Hofbauer cells were fetal origin macrophages (Figure S1E), highly expressed *FOLR2*, *CD45*, *FPLR2*, *CXCL8*, *AIF1*, *TYROBP*, *VSIG4*, *MRC1,* and *LYVE-1* (Figure 7A, Table S3). Meanwhile, another macrophage cluster robustly expressed *XIST* in each sample, and did not express *RPS4Y1*, which was of maternal origin (Figure S1E). Maternal macrophages might be derived from maternal blood in the intervillous space and attached to the outer layer of the placenta [72]. To further analyze the differences between early and mid-pregnancy in immune cells, DEGs and their GO analyses between 6–7 weeks of gestation and 14-16 weeks of gestation were explored (Figure 7B–E). Hofbauer cells in 6–7 weeks of gestation were characterized by cell proliferation and leukocyte activation (Figure 7C), whereas those in 14–16 weeks of gestation were associated with positive regulation of the MAPK cascade, cytokine production, and response to nutrient and oxygen levels (Figure 7C). These data showed that Hofbauer cells underwent a functional maturation transition from first to second trimester. Maternal macrophages also exhibited functional differences between early and mid-pregnancy (Figure 7E).

We performed pseudotime analysis to study the cell state transition process of erythroid cells (Figure 7F). State 3 branch was related to translational, ribosome biogenesis, and RNA catabolic processes, representing early stages of erythroid differentiation (Figure 7G,H). State 2 was associated with oxygen transport, and state 1 was associated with platelet degranulation and regulated exocytosis, which might represent erythroid or megakaryocytic lineage fates, respectively (Figure 7H). These results suggest that the erythroblasts we obtained from the first trimester placenta mainly include megakaryocyte-erythroid progenitors and cells in early erythroid differentiation stages. Interestingly, we found that cells in state 3 mostly existed in samples from 6–7 weeks of gestation (Figure 7I), and changed significantly into states 1 and 2 at approximately 9 weeks of gestation (Figure 7I).

Fibroblasts were divided into four subpopulations, referred to as F1-4 (Figure S7A). Cells in F1 specifically expressed *SFRP4*, *RORB*, *IL15*, and *MAOB* (Table S11), which act as regulators of adult uterine morphology and function [73,74,75,76]. F1 was mostly present in only one sample (Figure S7B), probably due to improper sampling. We considered F1 to be of maternal uterine origin and removed it in the subsequent analyses. The fibroblasts were thought to be a highly plastic cell population, with distinct gene expression patterns among different fibroblast clusters (Figure S7C,D).

## 4. Discussion

We used scRNA-seq to map the transcriptional landscape of human placentas from the first and second trimesters of pregnancy. Our study represents the first attempt to perform single-cell transcriptome analysis of human placental vascular development. We identified a population of endothelial progenitor cells (Endo-2) in the placenta. The other two endothelial cell clusters (Endo-1 and -3) were predominant at different stages of gestation and had distinct metabolic signatures. We proposed that Endo-1 might be involved in the formation of immature intervillous vascular beds in early pregnancy, while Endo-3 participates in active placental angiogenesis after the first trimester. Several signaling pathways (e.g., MAPK, TGF-β, ERBB, WNT, NOTCH, and VEGF signaling pathways) were activated in Endo-3 (Table S6). These results strongly suggested that the gene expression patterns of placental endothelial cells switch around the end of the first trimester, at which point more differentiated ECs enter the functional activation state.

High glycolytic activity has been demonstrated to be the primary source of ATP in ECs [54,77]. Most genes involved in glycolysis were upregulated in Endo-2, while *PFKFB3* was upregulated in Endo-3. Upon angiogenic stimuli, ECs nearly double their glycolytic flux, and this effect is mediated by an upregulation of *PFKFB3* [42]. The upregulation of *PFKFB3* in Endo-3 might be related to the proangiogenic environment and signal stimulation in the placenta. These results supported the idea that glycolysis plays an important role in both placental vascular progenitor cells and differentiated cells, with different expression patterns.

In the human placenta, the cytotrophoblasts differentiate along two major pathways, EVT differentiation and SCT differentiation. Our data pointed to the existence of two distinct populations of progenitor cells. We found that SCT progenitors (VCT-5) were inactively proliferating cells that existed in the inner layer of the villi. EVT progenitors (VCT-3) were actively proliferating cells located where the cell column begins to form, and maintained an EMT metastable phenotype. The presence of distinct progenitors for EVT and SCT may imply that placental VCTs should not be considered a direct stem cell population, whereas the true trophoblast stem cells are likely to be a less differentiated population and act as the precursor to both progenitor populations [1]. Previous studies reported several genes that mark the undifferentiated or stem-state VCTs, such as *TEAD4*, *TP63*, *YAP1,* and *ELF5* [16,78,79,80,81]. These genes were mainly present in VCT-1, VCT-2, and VCT-5 (Figure S5F). We also analyzed the functional differences of trophoblast between the first and second trimesters. We found that the functional enrichment of DEGs between mid- and early gestational stages was largely consistent across VCT subgroups and EVTs (Figures S8 and S9), with the early trophoblast dominated by basic life activities, such as cell proliferation, biosynthesis, and translation. In the second trimester, may be as maternal blood delivered into the placenta, all types of trophoblasts show inflammatory responses, response to cytokine stimulus or extracellular stimulus.

Interestingly, we found that 8–10 weeks of gestation is a dividing line for placental developmental status. Several lines of evidence support this conclusion: (1) The hierarchical clustering analysis showed similar transcriptome phenotypes of samples before 8 weeks of gestation in several placental cell types (Figure 1D); (2) the abundance of G2/M-phase ECs and trophoblasts decreased significantly after 8 weeks of gestation (Figure S2H, Figure S4G); (3) erythroblasts showed distinct differentiation characteristics in the sample at 9 weeks of gestation (Figure 7I); (4) after 9 weeks of gestation, the fibroblast subgroup with proangiogenic function appeared and became the dominant fibroblast subtype in the second trimester (Figure S7B); (5) a considerable increase in the proportion of ECs in the active angiogenic state was observed since 11 weeks of gestation (Figure 2C). This dividing line for placental developmental status overlaps with the time point when maternal blood flow to the placenta begins [10,82], prompting us to consider that this physiological change has a significant impact on placental development.

## 5. Conclusions

In summary, our study has provided a high-resolution molecular profile of human placental development between 6 and 16 weeks of gestation using scRNA-seq analysis. We analyzed the cellular subtype complexity within each placental cell type. We validated the heterogeneity of the placental endothelial cells to better understand the molecular determinants of normal placental endothelial function and activity. We identified two distinct populations of progenitor cells among VCTs that differentiated into EVTs and SCTs, respectively. These findings advance our understanding of the molecular and cellular mechanisms that regulate human placental development.

## Data Availability

The raw sequence data reported in this paper have been deposited in the National Genomics Data Center, China National Center for Bioinformation (GSA: HRA003309). The data that support the findings of this study are available from the corresponding author upon reasonable request.

## Supplementary Materials

The following supporting information can be downloaded at: https://www.mdpi.com/article/10.3390/cells12010087/s1, Figure S1. Characteristics of samples included in this study. (A) Violin plots of the number of UMI per cell (left), number of genes per cell (middle), and mitochondrial transcript ratio (right) in each cell type. (B) Gene expression patterns of each cell type. (C) Cell composition of the eleven samples. (D) A violin plot of *RPS4Y1* transcript expression in each sample for SCT, VCT, and EVT clusters. Samples with *RPS4Y1* expression were obtained from male fetuses. (E) A violin plot of *RPS4Y1* and *XIST* transcript expression in each sample for endothelial cells, fibroblasts, Hofbauer cells, and macrophages. Clusters with *XIST* expression only in D2, D4, D5, D9, and D10 are of fetal origin. Clusters with *XIST* expression in samples other than D2, D4, D5, D9, and D10 were of maternal origin. Figure S2. Single cell RNA-seq analysis of placental endothelial cells. (A) UMAP plots show the expression of the indicated marker genes in three placental vascular endothelial cell clusters. (B) A heatmap of gene expression levels of an endothelial cell differentiation-related gene set in three EC clusters. (C) A heatmap of gene expression levels of an endothelial cell migration-related gene set in three EC clusters. (D) Expression levels of vessel-type genes (artery, vein, and capillary) in three EC clusters. (E) Expression levels of endothelial cell location signature gene set (macrovascular and microvascular) in three EC clusters. (F) Heatmap of gene expression levels of tip cell markers in three EC clusters. (G) GSVA results of each ECs subgroup. Red represents upregulated pathways; blue represents downregulated pathways. (H) Cell cycle analysis of total endothelial cells at each developmental time. Figure S3. Pseudotime, SCENIC, and ligand-receptor interaction analysis in placental endothelial cell clusters. (A) Pseudotime analysis of placental endothelial cells, cells on the tree are colored by gestational age. (B) Regulon specificity score of the top 20 genes in each EC cluster. (C) Ligand-receptor interaction analysis within endothelial cell subpopulations and other placental cell types. Molecule 1 is expressed by Cell 1, and Molecule 2 is expressed by Cell 2. Figure S4. Single cell RNA-seq analysis of placental trophoblast cells. (A) UMAP plots showing expression of the representative marker genes in VCT, SCT, and EVT. (B) Unsupervised clustering of all trophoblast cells. (C-D) Eighteen unsupervised clustering groups were regrouped and merged by gene expression similarity, and eight subclusters were ultimately obtained. (E) Heatmap of the top differentially expressed genes of eight trophoblast subtypes. (F) Distribution of eight trophoblast subtypes in each sample. (G) Cell cycle analysis of total trophoblasts at each developmental time. Figure S5. Cell type-specific gene signatures in trophoblast subtypes. (A) Violin plots showing the expression levels of EMT-related genes, cell cycle-related genes, and fusion-related genes in trophoblast subtypes. (B) Violin plots showing the expression levels of invasion-related genes in EVT-1 and EVT-2. (C,D) Reactome pathway analysis of differentially expressed genes of EVT1 (C) and EVT2 (D). The size of the circles represents the number of significantly enriched genes in each item. The *p*-value significance is shown as a gradient of color in each circle, from blue (less significant) to red (more significant). (E) The expression of NOTCH pathway ligands and receptor genes in trophoblast clusters. The color key, from blue to yellow, indicates low to high average gene expression, respectively. The dot size indicates the percentage of cells expressing a certain marker. (F) UMAP plots showing expression of the representative trophoblast stemness-related genes in trophoblast subtypes. Figure S6. Immunofluorescence staining for trophoblast clusters. (A) Immunofluorescence staining for Ki67 in the early first trimester placenta (6 weeks of gestation). (B) Immunofluorescence staining for Ki67 in the second trimester placenta (14 weeks of gestation). The proliferative cells were localized in the villous cytotrophoblast and in the cytotrophoblast cell columns. Scale bars, 50 μm. (C,D) Immunofluorescence staining for the indicated VCT-5 marker CLIP1 and trophoblast marker EGFR in 6 weeks of gestation placentas. The white arrowheads indicate EGFR^+^CLIP1^+^ cells. Scale bars, 20 μm. (E) Immunofluorescence staining for C1QA and HLA-G in the decidua of 14 weeks of gestation human placenta. The white arrowheads indicated C1QA^+^HLA-G^+^ cells. Scale bars, 20 μm. (F) Immunofluorescence staining for the indicated VCT-3 marker TAGLN in 6 weeks of gestation placenta. The white arrowheads indicated VCT-3 cells, which are TAGLN and EGFR positive, HLA-G negative. Scale bars, 50 μm. Figure S7. Single cell RNA-seq analysis of placental fibroblasts. (A) UMAP plot showing four subtypes of fibroblast cells. (B) Distribution of four fibroblast subtypes in each sample. (C,D) Heatmap and GO enriched terms of cell type differentially expressed genes of fibroblast subclusters. Figure S8. Functional differences of endothelial cells and trophoblasts between the first and second trimesters. (A–C) GO analysis of the DEGs between 6–7 weeks of gestation (6–7 W) and 14–16 weeks (14–16 W) for endothelial cells (A), VCT (B), and EVT (C). The size of the circles represents the number of significantly enriched genes in each item. The color key, from blue to red, indicates low to high average gene expression. Figure S9. Functional differences of trophoblast subgroups between the first and second trimesters. (A–C) Volcano plot of the DEGs between 6-7 weeks of gestation (6–7 W) and 14–16 weeks of gestation (14–16 W) for VCT-1 (A), VCT-2 (B), and VCT-5 (C). (D-F) Representative GO analysis terms for DEGs between 6–7 W and 14–16 W in VCT-1 (D), VCT-2 (E), and VCT-5 (F). Table S1. Primers used for qRT-PCR. Table S2. Sample information. Table S3. Cell-type specific signature genes. Table S4. Number of cells analyzed from each sample per cluster. Table S5. Differentially expressed genes among the three placental endothelial cell clusters. Table S6. Functional and pathway enrichment analysis for three placental endothelial cell clusters. Table S7. SCENIC regulon activity and specificity analysis in EC subtypes. Table S8. Expression levels of transcription factors in EC subtypes. Table S9. Differentially expressed genes among the eight placental trophoblast clusters. Table S10. Functional and pathway enrichment analysis of differentially expressed genes in EVTs. Table S11. Differentially expressed genes among fibroblasts subclusters.
