# Supplementary material for: Human Placental Endothelial Cell and Trophoblast Heterogeneity and Differentiation Revealed by Single-Cell RNA Sequencing
Source: Cells. 2022 Dec 25;12(1):87. doi: 10.3390/cells12010087 (PMC9818681; doi:10.3390/cells12010087)
Supplement: Supplementary file 1 [file cells-12-00087-s001.zip › Supplementary Table S1 Primers used for qRT-PCR.pdf]

Table S1. PCR primer sequences

Gene	Forward primer	Reverse primer
GAPDH	CGCTGAGTACGTCGTGGAGTC	GCTGATGATCTTGAGGCTGTTGTC
ERVFRD-1	AGCAGCCGTAGTCCTTCAAA	AGGGGAAGAACCCAAGAGAA
CGA	TTTCTGGTCACATTGTCGGT	TGGGCAATCCTGCACATCAG
CGB	CCTGGCCTTGTCTACCTCTT	CCTGGCCTTGTCTACCTCTT
TBX3	AAAAATAGACAACAACCCTTTTGC	ACTGCAGGGTGAGCTGTTTT
CLIP1	AGAAAGCAAGCCGACAAA	CTCCAAGGAGGCCAGAGT
