# Supplementary figures and images for: Human Placental Endothelial Cell and Trophoblast Heterogeneity and Differentiation Revealed by Single-Cell RNA Sequencing
Source: Cells. 2022 Dec 25;12(1):87. doi: 10.3390/cells12010087 (PMC9818681; doi:10.3390/cells12010087)

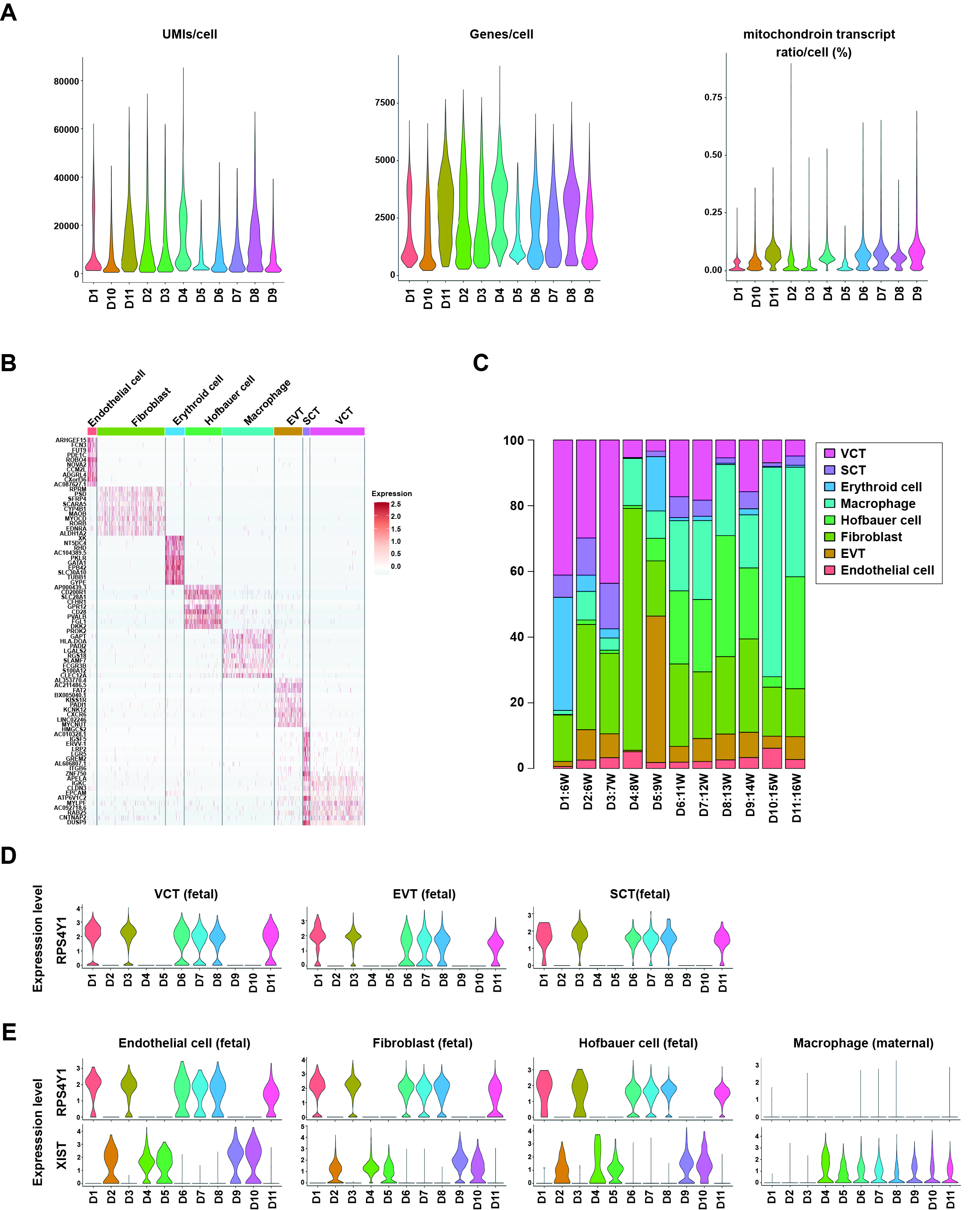

Supplement: Supplementary file 1 [file cells-12-00087-s001.zip › supplementary Figure S1.tif]

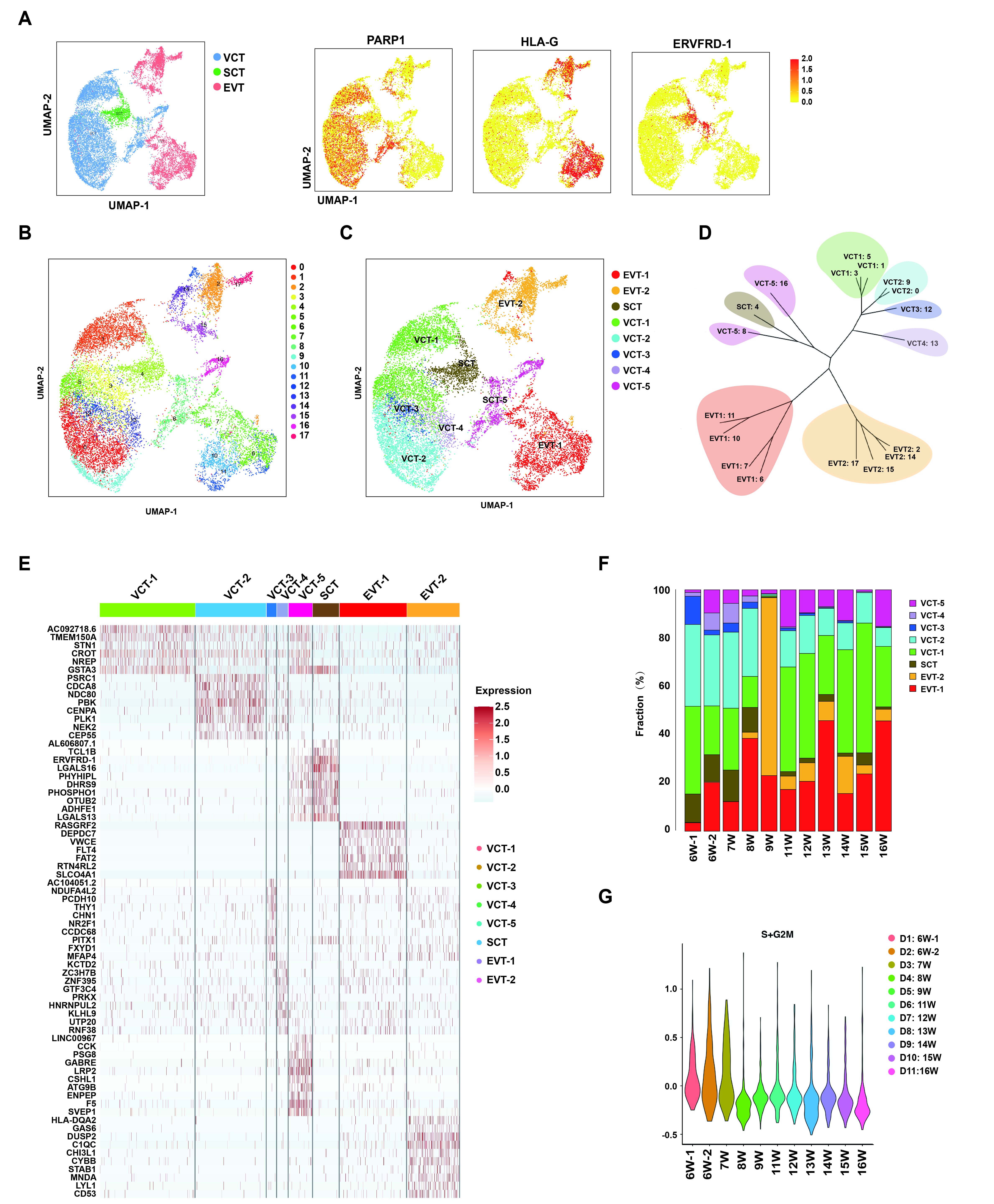

Supplement: Supplementary file 1 [file cells-12-00087-s001.zip › supplementary Figure S4.tif]

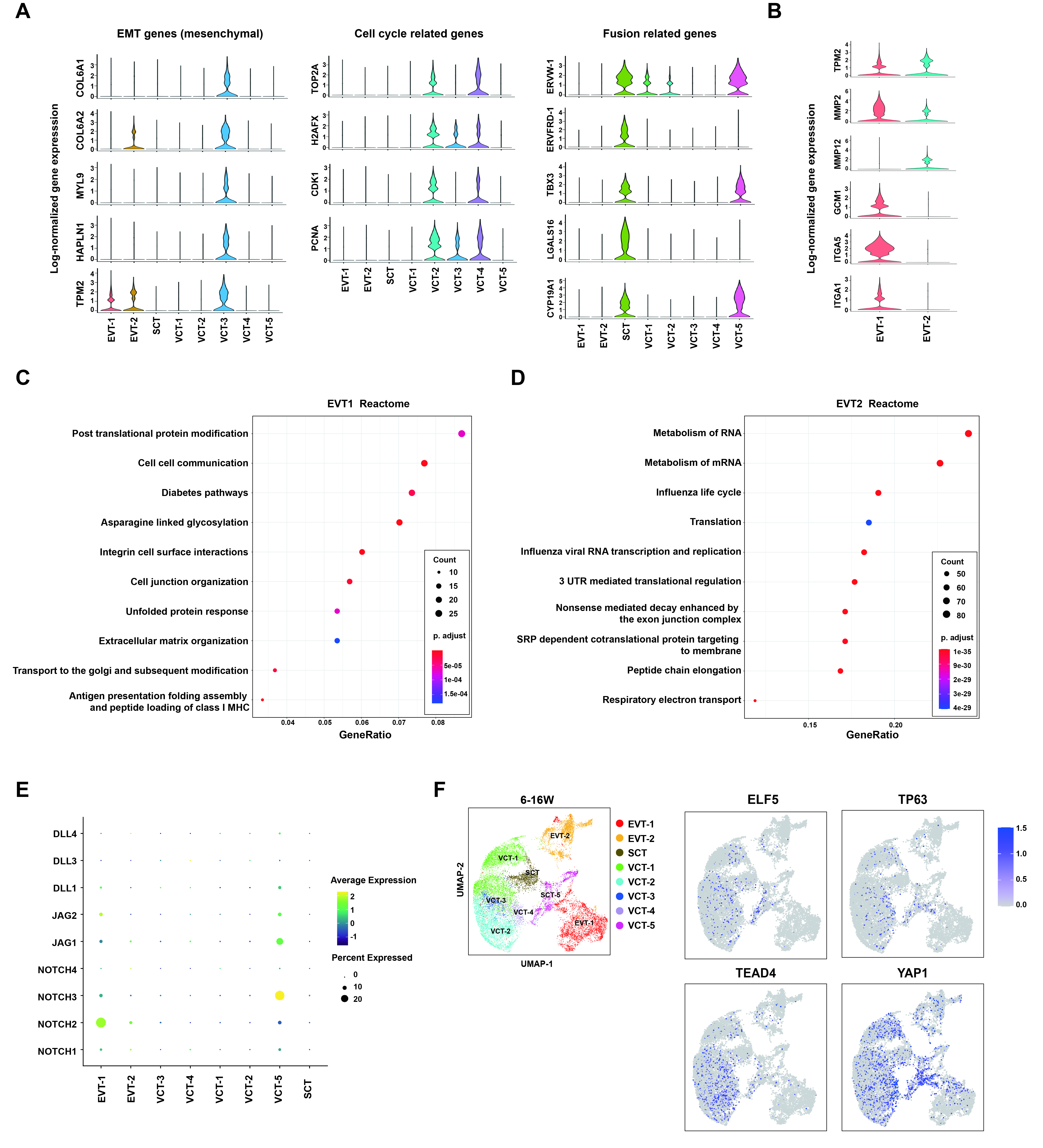

Supplement: Supplementary file 1 [file cells-12-00087-s001.zip › supplementary Figure S5.tif]

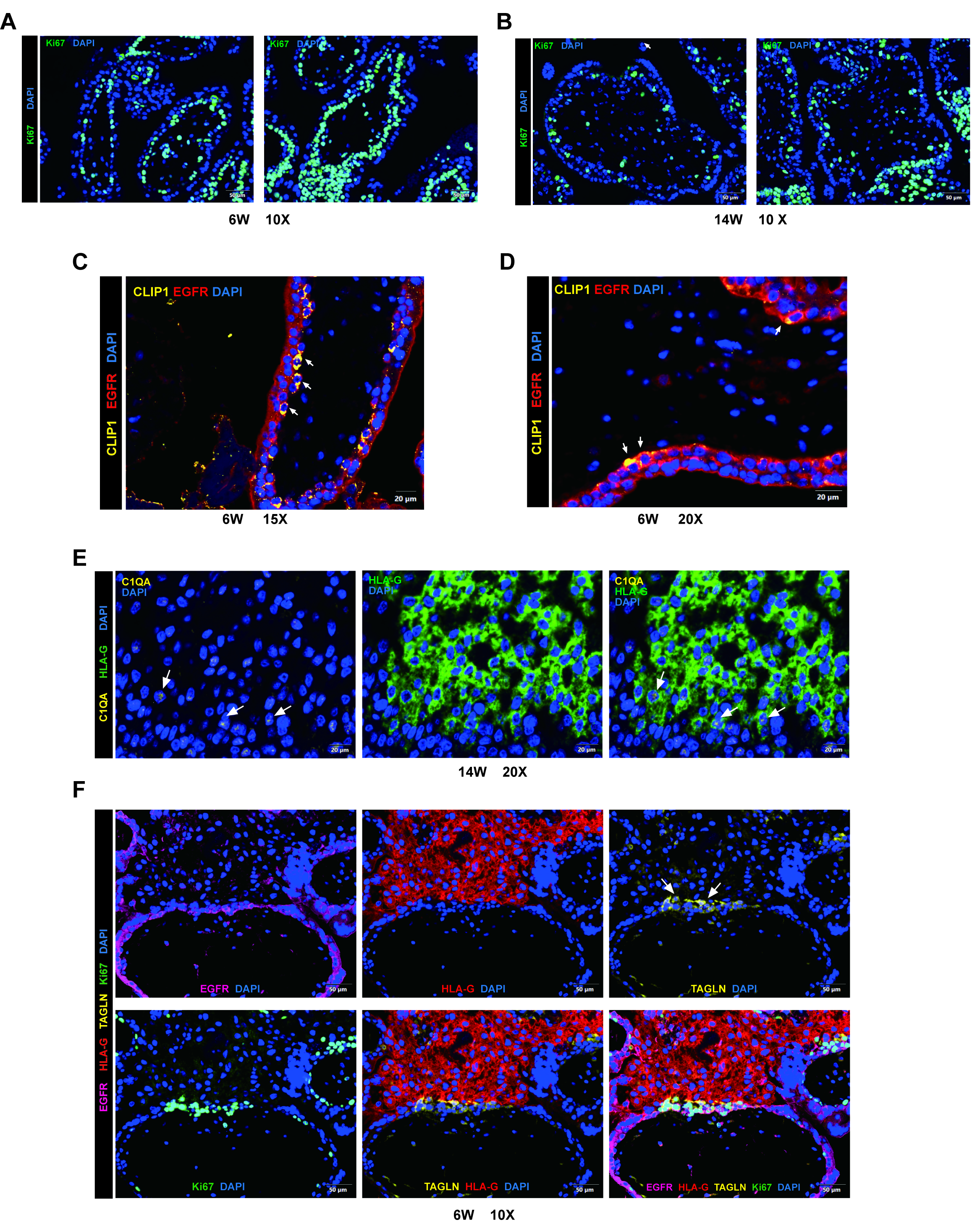

Supplement: Supplementary file 1 [file cells-12-00087-s001.zip › supplementary Figure S6.tif]

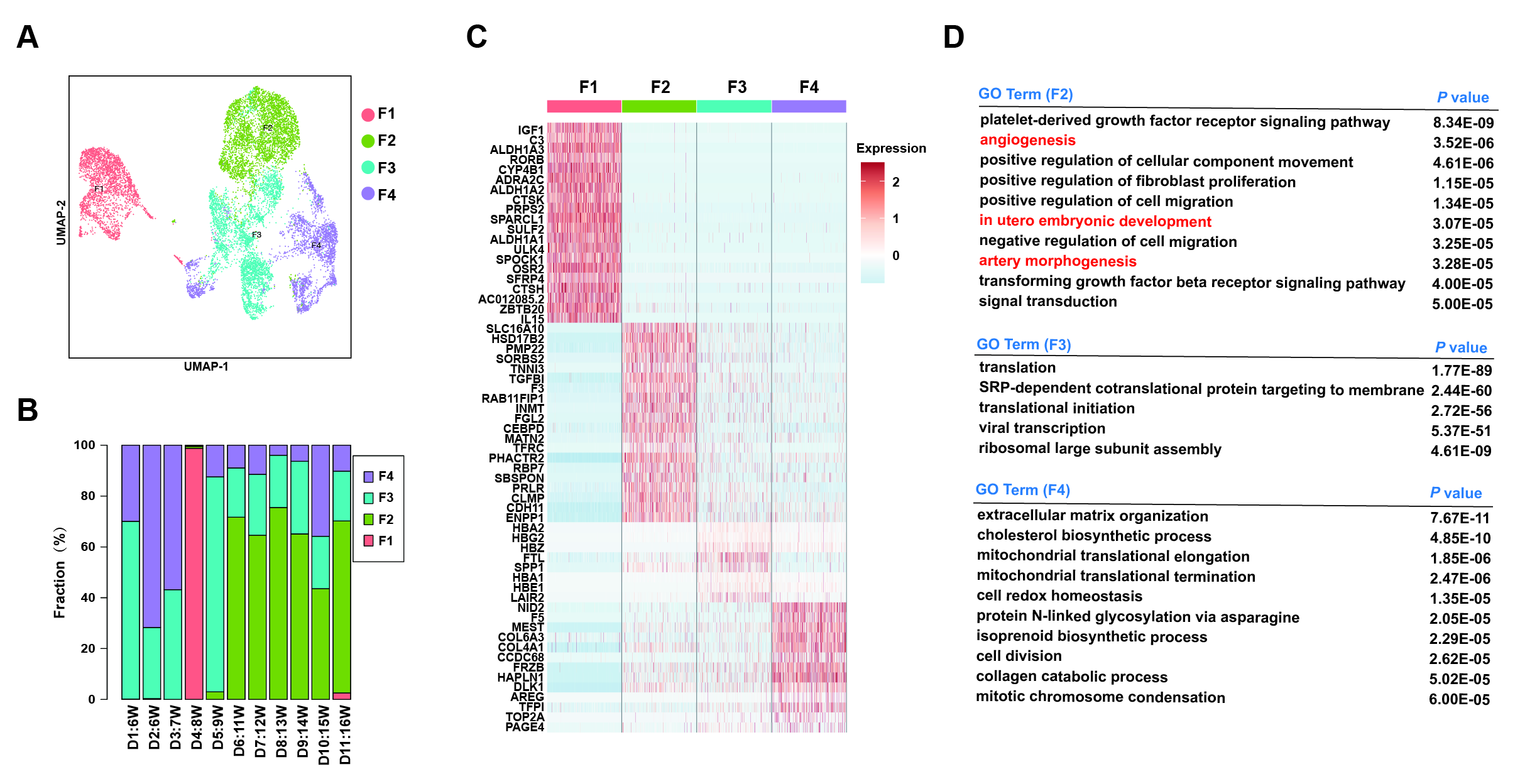

Supplement: Supplementary file 1 [file cells-12-00087-s001.zip › supplementary Figure S7.tif]

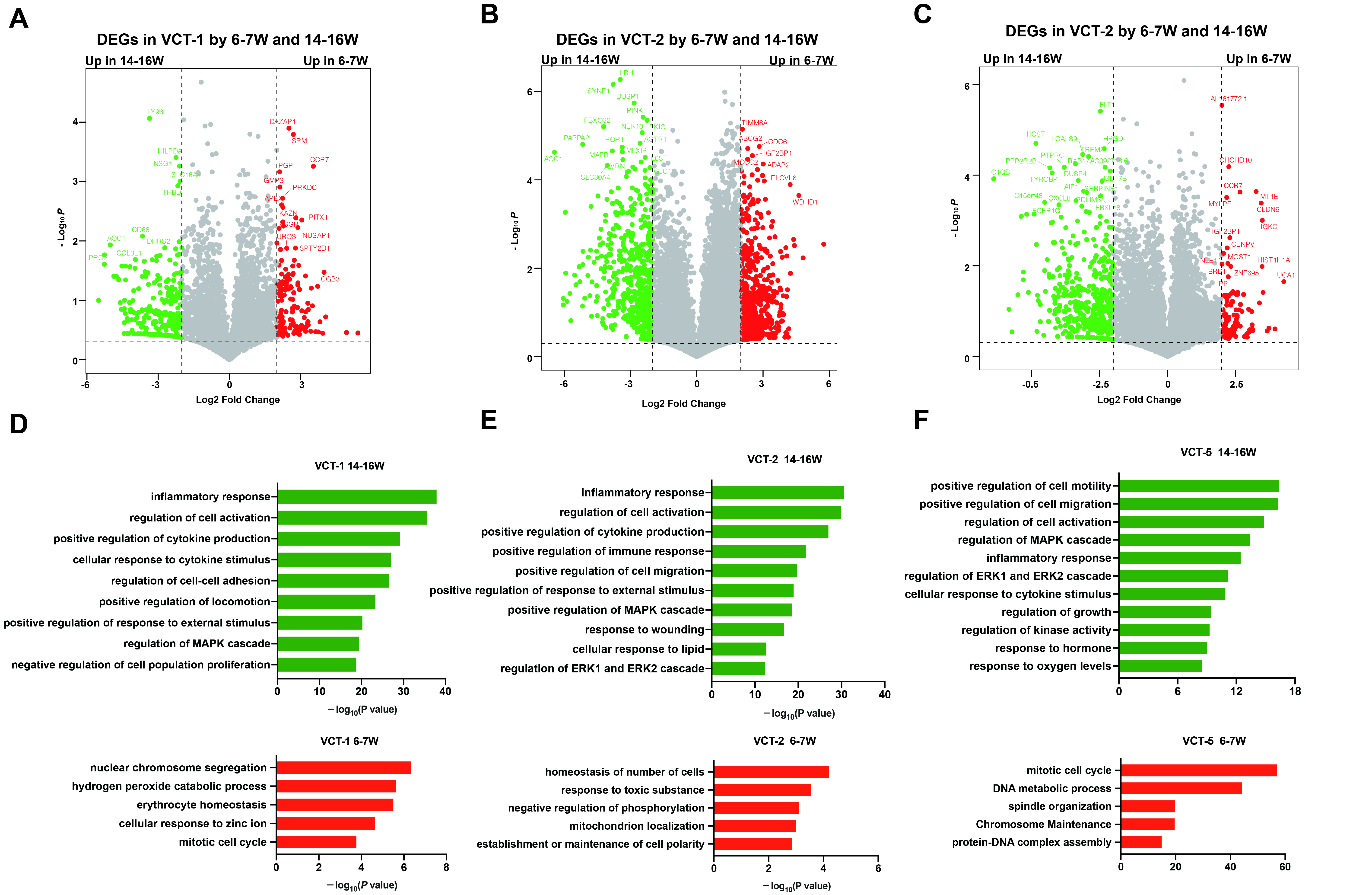

Supplement: Supplementary file 1 [file cells-12-00087-s001.zip › supplementary Figure S9.tif]
